# Partial Clinical Remission Reduces Lipid-Based Cardiovascular Risk in Adult Patients With Type 1 Diabetes

**DOI:** 10.3389/fendo.2021.705565

**Published:** 2021-11-17

**Authors:** Benjamin Udoka Nwosu, Sadichchha Parajuli, Krish Khatri, Gabrielle Jasmin, Layana Al-Halbouni, Austin F. Lee

**Affiliations:** ^1^ Division of Endocrinology, Department of Pediatrics, University of Massachusetts Medical School, Worcester, MA, United States; ^2^ Division of Endocrinology, Department of Internal Medicine, University of Massachusetts Medical School, Worcester, MA, United States; ^3^ Department of Population and Quantitative Health Sciences, University of Massachusetts Medical School, Worcester, MA, United States

**Keywords:** type 1 diabetes, type 2 diabetes, adults, honeymoon phase, partial clinical remission, cardiovascular disease risk, dyslipidemia

## Abstract

**Importance:**

Risk factors for atherosclerotic cardiovascular disease (ASCVD) are well established in type 2 diabetes (T2D), but not in type 1 diabetes (T1D). The impact of partial clinical remission (PR) on short-term ASCVD risk in T1D is unclear.

**Aim:**

To investigate the impact of PR on the earliest ASCVD risk phenotype in adult T1D using factor analysis to compare the lipid phenotypes of T1D, T2D and controls after stratifying the T1D cohort into remitters and non-remitters.

**Subjects and Methods:**

A study of 203 adults subjects consisting of 86 T2D subjects, and 77 T1D subjects stratified into remitters (n=49), and non-remitters (n=28). PR was defined as insulin-dose adjusted HbA1c of ≤9, and obesity as a BMI ≥30 kg/m^2^. Factor analysis was used to stratify the groups by ASCVD risk by factorizing seven lipid parameters (TC, LDL, HDL, non-HDL, TC/HDL, TG, TG/HDL) into 2 orthogonal factors (factor 1: TC*LDL; factor 2: HDL*TG) that explained 90% of the variance in the original seven parameters.

**Results:**

The analysis of individual lipid parameters showed that TC/HDL was similar between the controls and remitters (p=NS) but was significantly higher in the non-remitters compared to the remitters (p=0.026). TG/HDL was equally similar between the controls and remitters (p=NS) but was lower in the remitters compared to the non-remitters (p=0.007). TG was significantly lower in the remitters compared to T2D subjects (p<0.0001) but was similar between T2D subjects and non-remitters (p=NS). Non-HDL was significantly lower in the controls *versus* non-remitters (p=0.0003) but was similar between the controls and remitters (p=NS). Factor analysis showed that the means of factor 1 and factor 2 composite scores for dyslipidemia increased linearly from the controls, remitters, non-remitters to T2D, p value 0.0042 for factor 1, and <0.0001 for factor 2, with remitters having similar lipid phenotype as controls, while non-remitters were similar to T2D.

**Conclusions:**

Partial clinical remission of T1D is associated with a favorable early lipid phenotype which could translate to reduced long-term CVD risk in adults.

## Introduction

Diabetes mellitus affects 34.2 million Americans, or 10.5% of the population (1), and atherosclerotic cardiovascular disease (ASCVD) is the leading cause of death in individuals with diabetes (1). Risk factors for ASCVD are well established in type 2 diabetes (T2D), but not in type 1 diabetes (T1D) (1), and the impact of partial clinical remission (PR) on long-term ASCVD in T1D is unclear.

The major link between diabetes mellitus and ASCVD, referred to as diabetic dyslipidemia, occurs in the setting of low high-density lipoprotein cholesterol (HDL-C), high fasting and/or postprandial triglycerides (TG), average to high low-density lipoprotein cholesterol (LDL-C), and predominantly small dense LDL particles (2). Additionally, elevated non-HDL-C correlates with 99% increased CVD risk for patients with T2D (3) where the classic lipid abnormalities are characterized by elevated TG, small dense LDL-C, and low HDL-C (4). CVD risk in patients with T1D is predicted by total cholesterol/HDL cholesterol (TC/HDL), and non-HDL cholesterol but not LDL-C (5).

Despite the establishment of the correlation between ASCVD and diabetes, the underlying mechanisms remain poorly understood (6), especially in T1D where 50% of the subjects undergo partial clinical remission (PR) or honeymoon phase following the diagnosis of T1D (7). PR usually lasts for 3-12 months (8), but could be longer (9).

However, the impact of PR on the earliest lipid phenotypes in adults with diabetes mellitus is not known as no study has compared the earliest lipid phenotypes of remitters (those who underwent partial clinical remission) and non-remitters (those who did not undergo partial clinical remission) to controls and their peers with T2D. This is important because studies in children and emerging adults have reported that PR modulates the degree of early-phase dyslipidemia (10), mid-term microvascular disease risk (11), and long-term CVD risk (12). Secondly, factor analysis (13, 14) has not been employed to investigate the relationships between primary and secondary lipid parameters in order to reduce them to fewer underlying summary factors to enable precise stratification of ASCVD risk among the remitters, non-remitters, subjects with T2D and controls.

The aim was to investigate the impact of PR on the earliest ASCVD risk phenotype in adult T1D using factor analysis to compare the lipid phenotypes of T1D, T2D and controls after stratifying the T1D cohort into remitters and non-remitters. This aim focused primarily on the factor analysis of the ADA-recommended initial lipid parameters for the assessment of CVD risk in adults with diabetes namely, TG and HDL, and the atherogenic index of plasma, TG/HDL as factor 2; and secondarily on non-HDL-C, LDL-C, TC, TC/HDL ratio as factor 1. We hypothesized that remitters, non-remitters, and subjects with T2D will have similar lipid phenotype when compared to controls.

## Materials and Methods

### Ethics Statement

The Institutional Review Board of the University of Massachusetts approved the study protocol and the waiver of authorization to review subjects’ retrospective records under Docket # H00015476. All subjects’ data were anonymized and de-identified prior to analysis.

### Subjects

The patient population consisted of 203 adult patients from the Adult Diabetes Clinic Database of the UMassMemorial Medical Center, Worcester, Massachusetts, USA. This study was a comparative analysis of anthropometric and biochemical data in the first year of either T1D or T2D in 86 subjects with T2D of age 45.0 ± 10.5 y; and 77 subjects with T1D stratified into remitters (n = 49, age 29.7 ± 10.0 y) and non-remitters (n=28, age 31.9 ± 11.0 y).

Male and female subjects of 18-65 years were included in the study if they had a diagnosis of either T1D or T2D and were not on statin therapy and had no other systemic disease apart from diabetes mellitus. The control group consisted of healthy adults who were seen for routine physical examination.

Subjects and their time of diagnosis of diabetes mellitus were identified from the electronic medical records using the International Classification of Diseases (ICD-10) codes for T1D and for T2D. Subsequently, each subject’s record was manually reviewed for verification of diagnosis of T1D and T2D, and for the collection of anthropometric and biochemical data. The criteria for the diagnosis of T1D and T2D have been previously described in detail (10, 15–18). Briefly, the diagnosis of T1D and T2D was established based on any of the following glycemic indices: a fasting blood glucose of ≥ 126 mg/dL (7 mmol/L), and/or 2-hour postprandial glucose of ≥200 mg/dL (11.1 mmol/L), and/or random blood glucose of ≥200 mg/dL with symptoms of polyuria and/or polydipsia. The establishment of a diagnosis of T1D further required the detection of one or more diabetes-associated auto-antibodies, namely islet cell cytoplasmic-, insulin-, insulinoma-associated-2-, zinc transporter 8, and glutamic acid decarboxylase autoantibodies. Individuals diagnosed with other forms of diabetes mellitus were excluded from the study. Such individuals could have developed pancreatic insufficiency from cystic fibrosis, surgeries, and pancreatitis. Pregnant patients were also excluded from this study.

Anthropometric, glycemic, and lipid data were collected at the time of diagnosis of either T1D or T2D or during the first clinic visit following the diagnosis that occurred within the first 2 weeks following the diagnosis of diabetes mellitus. All lipid data for this study were obtained within the first month of the diagnosis of diabetes mellitus. Data on glycemic control were obtained at diagnosis, and at 6 months and 12 months to establish a longitudinal pattern of glycemic control, and for the verification of PR in the first year following the diagnosis of diabetes mellitus.

PR was defined by insulin dose-adjusted hemoglobin A1c (IDAA1c) (8), a formula that integrates both HbA1c and total daily dose of insulin (TDDI), and is expressed as HbA1c (%) + [4 X total daily dose of insulin (units/kg/24h)] (8). The presence of PR was denoted by IDAA1c of ≤9. IDAA1c is a surrogate marker of the gold-standard definition of PR, which is either a fasting C-peptide level of >0.1 nmol/L (0.3 ng/mL) (19) or a 2-hour post-meal stimulated C-peptide level of 0.2 nmol/L (≥0.6 ng/mL) (20). Subjects characterized as remitters had IDAA1c of ≤9 in the first year, while the non-remitters never attained an IDAA1c of ≤9 throughout the one-year period of observation.

The total daily dose of insulin was obtained directly from the electronic medical records from scanned copies of insulin pump data downloads, or from documented insulin doses in physicians’ notes.

### Anthropometry

Body mass index (BMI) was calculated from the formula: weight/height^2^ (kg/m^2^). Obesity was defined by a BMI ≥30 kg/m^2^ and non-obese as BMI <30 kg/m^2^.

### Assays

The assay methodologies have been previously described (10, 16, 21, 22). Serum lipid analysis was performed at the University of Massachusetts Medical School Clinical Laboratory based on the Beckman Coulter AU system that meets the National Cholesterol Education Program’s criteria for accuracy (23). Serum LDL-cholesterol concentration was calculated by the Friedewald equation when triglyceride levels were <400 mg/dL (24), and by the beta quantification procedure for triglyceride levels of ≥ 400 mg/dL (25). Assays for diabetes-associated autoantibodies were performed by Quest Diagnostics, Chantilly, VA, USA. Specifically, GAD-65 assay was conducted using enzyme-linked immunosorbent assay, and IA-2A and IAA assays were processed using radio-binding assay.

### Statistical Analyses

Means and standard deviations (SD) were calculated for continuous variables and biochemical parameters. ANOVA was used to compare the study groups: controls, remitters, non-remitters, and T2D. For categorical variables, numbers (n) and percentages (%) were compared using contingency-table Chi-square test. Remission status was defined by an IDAA1c of ≤9, and non-remission by an IDAA1c of >9 (8). Data on lipid parameters and ratios: TC, LDL-C, HDL-C, TC/HDL, non-HDL-C, TG, and TG/HDL were presented as box and whisker plots. Since distributions of these parameters were skewed, regression models were performed on their median values for group comparisons, and adjusted for age, sex, race, and obesity (defined by a BMI ≥30 kg/m^2^). Scheffe’s method was used for pairwise comparisons of median values of lipid parameters among the study groups. In anticipating high correlations between certain pairs of lipid parameters, we performed factor analysis on 7 lipid parameters using Varimax rotation to obtain composite scales that were orthogonal to (uncorrelated with) each other. We displayed factor loadings, variance explained, and communality to investigate the achievement of the dimension-reduction process. The distributions of the lipid-composite scores were presented as box and whisker plots. Ordinary least square (OLS) regression models, adjusted for age, sex, race, and body mass index, were used to ascertain linear trend of the mean lipid-composite scores across study groups. Furthermore, the change in the means of composite scores across study groups were characterized on a two-dimensional scale for validity. A p-value of <0.05 was considered statistically significant in all cases. All analyses were performed using SAS 9.4 software (SAS Institute Inc, Cary, NC).

## Results

### General Parameters

We analyzed the data of 203 subjects consisting of 40 controls, 77 subjects with T1D, and 86 subjects with T2D. The subjects with T1D were further divided into remitters (n=49) and non-remitters (n=28). The overall mean age was 37.3 y ( ± 12.7 SD), with males 51.7% and whites 71.3%. **Table 1** shows the baseline anthropometric and biochemical characteristics of the subjects by study groups. There were no significant differences in height or gender distribution between the remitters, non-remitters, and subjects with T2D (p=0.44 and 0.91, respectively). Subjects with T2D were older, heavier, and had higher systolic and diastolic blood pressure readings than the subjects with T1D (p<.0001). The non-remitters had significantly higher fasting blood glucose levels (p<.0001).

**Table 1:** Comparison of anthropometric, biochemical, and therapeutic parameters.

Parameters	Controls (n=40)		Remitters (n=49)		Non-remitters (n=28)		Type 2 diabetes (n=86)		ANOVA F-test p value		
	Mean	SD	Mean	SD	Mean	SD	Mean	SD	All 4 groups	3 DM groups	
Age (year)	33.8	11.0	29.7	10.9	31.9	11.0	45.0	10.5	<.0001	<.0001	
Height (cm)	164.9	9.5	170.9	11.8	167.1	8.8	170.4	10.2	0.0450	0.44	
Weight (kg)	74.8	20.2	78.6	17.4	68.6	11.3	104.7	28.7	<.0001	<.0001	
BMI (kg/m^2^)	26.6	6.1	27.0	6.3	25.6	3.2	35.4	9.5	<.0001	<.0001	
SBP (mm Hg)	114.6	17.6	117.5	13.0	113.3	14.0	133.3	17.3	<.0001	<.0001	
DBP (mm Hg)	71.4	13.9	74.5	8.2	70.7	10.7	83.5	10.8	<.0001	<.0001	
FBS (mg/dL)			224	130	425	232	201	116		<.0001	
TC (mg/dL)	155.8	18.6	182.6	72.1	186.2	49.1	192.5	44.9	0.0014	0.62	
LDL-C (mg/dL)	86.1	17.9	100.5	34.8	105.1	32.1	110.9	35.0	0.0011	0.31	
HDL-C (mg/dL)	55.6	12.4	50.5	13.8	46.7	12.8	38.8	9.8	<.0001	<.0001	
TC/HDL	2.9	0.7	4.1	3.9	4.2	1.6	5.2	1.7	<.0001	0.0329	
Non-HDL-C (mg/dL)	100.1	20.3	132.1	74.3	139.5	48.6	153.7	45.6	<.0001	0.11	
TG (mg/dL)	70.4	26.5	120.4	86.0	171.1	168.1	256.6	277.2	<.0001	0.0095	
TG/HDL	1.3	0.6	2.7	2.3	4.2	4.7	7.6	9.5	<.0001	0.0041	
HbA1c at 0 mo (%)			11.6	2.4	11.7	2.5	8.8	2.3		<.0001	
HbA1c at 6 mo			6.5	0.9	9.0	2.1	7.1	1.4		<.0001	
HbA1c at 12 mo			6.8	1.3	9.4	2.4	7.4	1.7		<.0001	
TDD at baseline(units/kg/day)			0.39	0.18	0.51	0.29	0.40	0.18		0.15	
TDD at 6 mo			0.39	0.20	0.70	0.35	0.27	0.19		<.0001	
TDD at 12 mo			0.42	0.21	0.81	0.31	0.33	0.29		<.0001	
Metformin (mg) baseline							1069	501			
Metformin (mg) final							1492	548			
	n	%	n	%	n	%	n	%			
Sex											
Male	12	30.0	28	57.1	15	53.6	50	58.1	0.0224	0.91	
Female	28	70.0	21	42.9	13	46.4	36	41.9			
Race/Ethnicity											
White	26	65.0	44	91.7	18	64.3	56	65.1	0.0051*	0.0022*	
Black	6	15.0	1	2.1	1	3.6	10	11.6			
Asian	3	7.5	0	0.0	1	3.6	4	4.7			
Hispanic	5	12.5	2	4.2	8	28.6	14	16.3			
Other	0	0.0	1	2.1	0	0.0	2	2.3			

BMI, body mass index; SBP, systolic blood pressure; DBP, diastolic blood pressure; TC, total cholesterol; TG, triglycerides; HDL, high density lipoprotein cholesterol; LDL, low density lipoprotein cholesterol; HbA1c, hemoglobin A1c; mo,month; TDD, total daily dose of insulin. *p value for white versus others.

### Parameters of Glycemic Control

At diagnosis, mean HbA1c was significantly higher in the remitters (11.6 ± 2.4%) and non-remitters (11.7 ± 2.5%) compared to the subjects with T2D (8.8 ± 2.3%) (p<0.0001). However, at 6 months, A1c was significantly higher in the non-remitters (9.0 ± 2.1%), compared to the remitters (6.5 ± 0.9%) and the subjects with T2D, (7.1 ± 1.4%) (p<0.0001). A similar pattern was also present at 12 months, with the non-remitters showing significantly higher A1c values compared to the remitters and T2D subjects, (p<0.0001).

### Lipid Analysis

#### Analysis of Individual Lipid Parameters and Ratios

We first analyzed the changes in the individual and composite lipid parameters. For the individual lipid parameters, we reported the median and the first and the third quartiles to address the skewed distribution of these parameters.

#### Non-HDL-C

**Figure 1:**
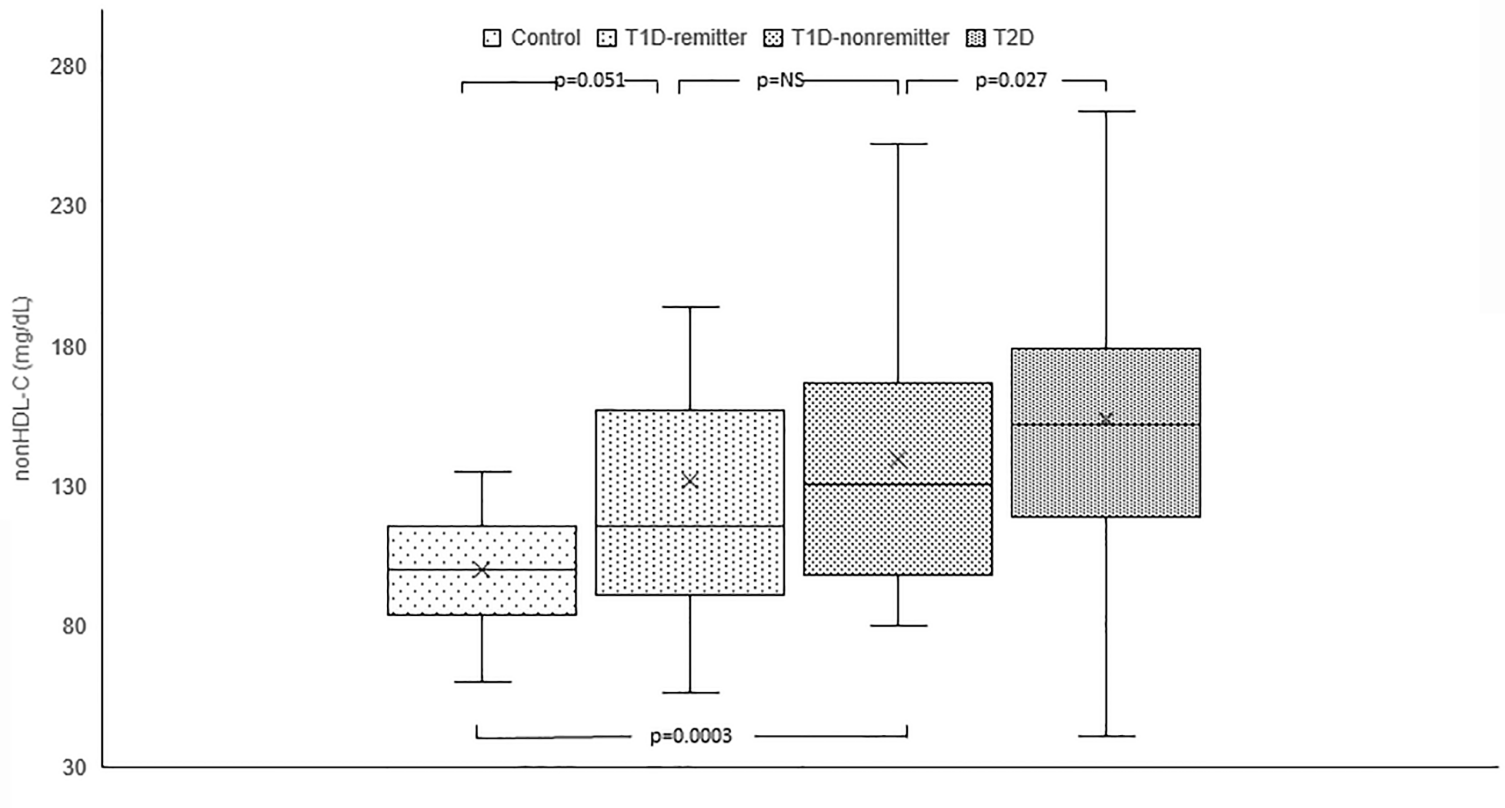
Box plots of early post-diagnostic patterns of non-high density lipoprotein cholesterol (non-HDL-C) in the remitters, non-remitters, and subjects with type 2 diabetes (T2D) compared to controls. The box represents the 50th percent interquartile range, while the ‘x’ represents the mean and the horizontal line within the box represents the median, and the upper and lower whiskers represent 25^th^ percentile above and below the mean, respectively.

Serum non-HDL was significantly lower in the controls [median=100 mg/dL, Q1-Q3= (84-116)] compared to the subjects with T2D (152 mg/dL, 119-179, p<0.0001), and the non-remitters (131 mg/dL, 100-167, p<0.0001); but was similar to the remitters (116 mg/dL, 92-155, p=0.051). Additionally, non-HDL was significantly lower in the non-remitters compared to the subjects with T2D (p=0.027) but was similar between the remitters and non-remitters (p=0.39) (**Figure 1**).

#### TG

**Figure 2:**
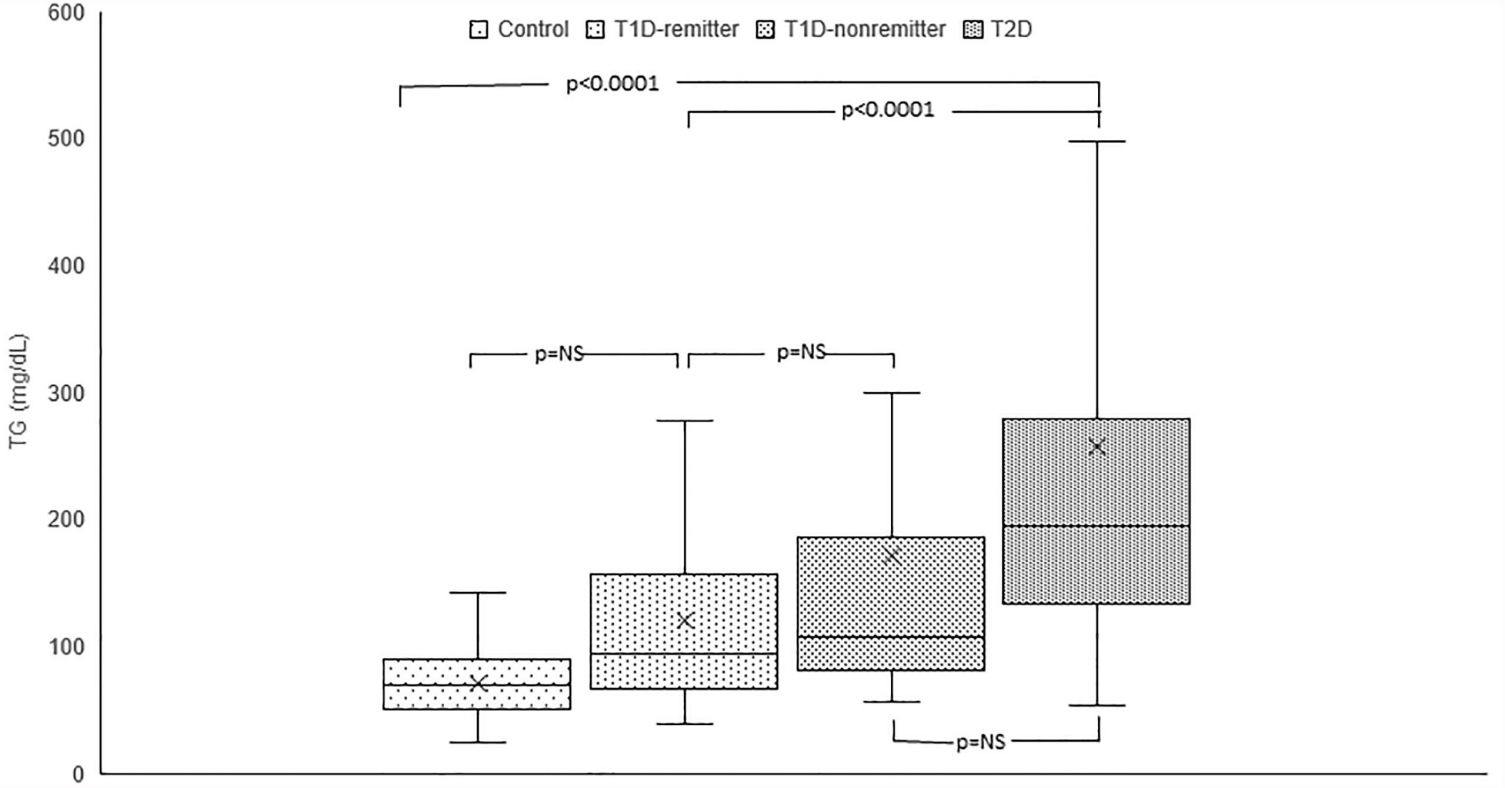
Box plots of early post-diagnostic patterns of triglycerides (TG) in the remitters, non-remitters, and subjects with type 2 diabetes (T2D) compared to controls.

Serum TG concentration was significantly lower in the controls (69 mg/dL, 50-88) compared to the subjects with T2D (194 mg/dL, 134-276, p<0.0001) but was similar between the remitters and non-remitters (94 mg/dL, 66-157 *vs* 107 mg/dL, 82.5-184, p=NS). However, while TG was similar between the non-remitters and subjects with T2D (p=NS), it was significantly lower in the remitters compared to the subjects with T2D (p<0.0001). (**Figure 2**).

#### TG/HDL

**Figure 3:**
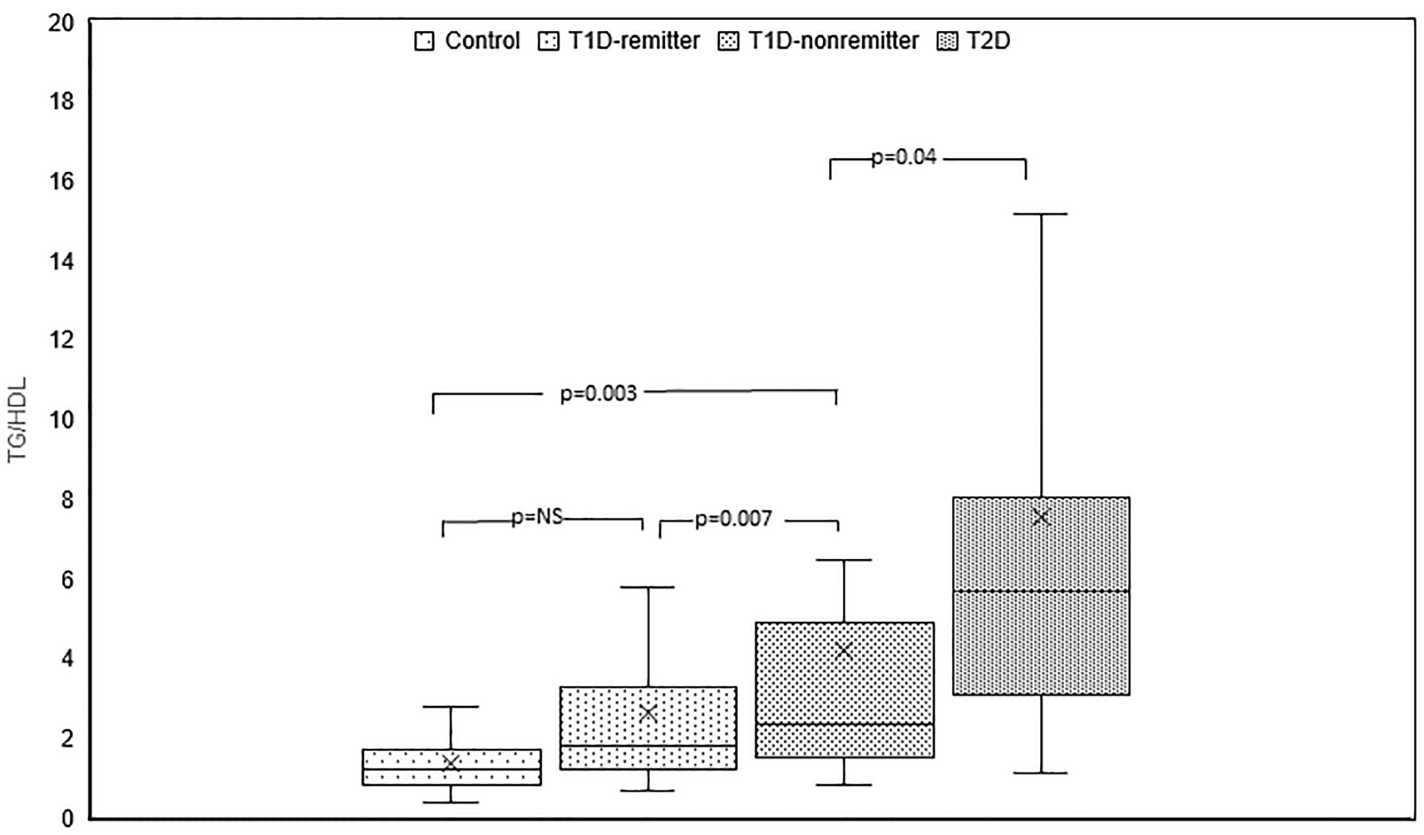
Box plots of early post-diagnostic patterns of triglycerides/high-density lipoprotein cholesterol ratio (TG/HDL) in the remitters, non-remitters, and subjects with type 2 diabetes (T2D) compared to controls.

TG/HDL ratio was significantly different among the 4 groups (p<0.0001). *Post hoc* analysis showed that TG/HDL ratio was significantly lower in the controls compared to the subjects with T2D (1.2, 0.9-1.7 *vs* 5.7, 3.1-8, p<0.0001), the non-remitters (1.2, 0.9- 1.7 *vs* 2.4, 1.5-4.9, p=0.003), but similar to the remitters (1.2, 0.9-1.7 *vs* 1.8, 1.2-3.3, p=NS). Furthermore, TG/HDL was significantly lower in the remitters compared to the non-remitters (p=0.007) (**Figure 3**).

#### LDL-C

There was a significant difference in serum LDL among the 4 groups (p<0.0005). *Post hoc* analysis showed no difference in LDL levels between the remitters, non-remitters, and subjects with T2D. However, when compared to the controls, LDL was significantly higher in the subjects with T2D (p<0.0004), non-remitters (p=0.009), but similar to the remitters (p=0.052).

#### HDL

Serum HDL was significantly lower in the subjects with T2D compared to the controls (52.5 mg/dL, 45.5-67 *vs* 36 mg/dL, 31-45, p<0.0001), non-remitters (52.5 mg/dL, 45.5-67 *vs* 49.5 mg/dL, 34.5-56, p=0.0217), and remitters (52.5 mg/dL, 45.5-67 *vs* 47.5, 42-62, p<0.0001). HDL was similar between the non-remitters and remitters (49.5 mg/dL, 34.5-56 *vs* 47.5 mg/dL, 42-62, p=NS).

#### TC/HDL

**Figure 4:**
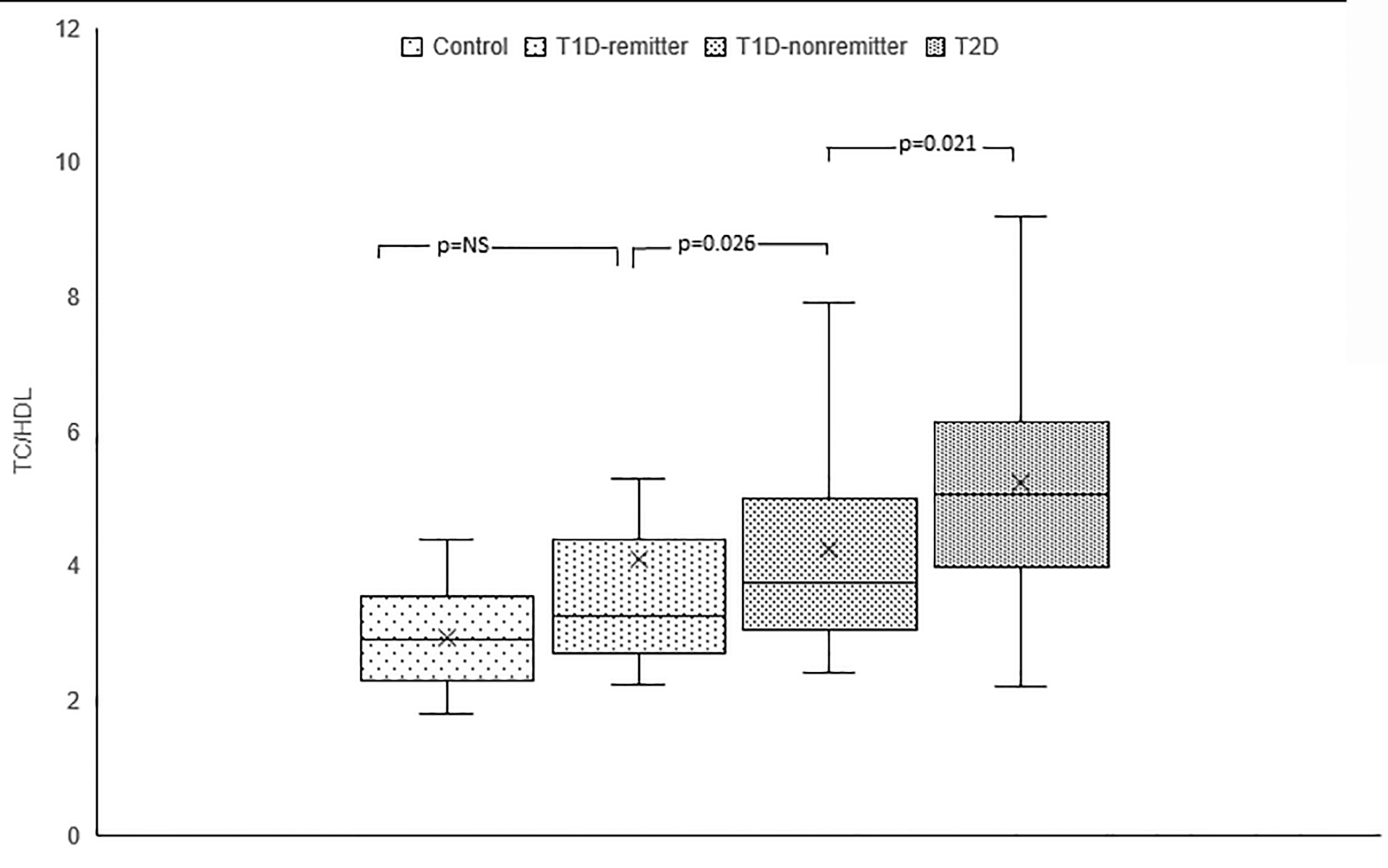
Box plots of early post-diagnostic patterns of total cholesterol/high-density lipoprotein cholesterol ratio (TC/HDL) in the remitters, non-remitters, and subjects with type 2 diabetes (T2D) compared to controls.

TC/HDL ratio was significantly lower in the controls compared to the subjects with T2D (2.9, 2.3-3.5 *vs* 5.1, 4-6.1, p<0.0001), the non-remitters (2.9, 2.3-3.5 *vs* 3.8, 3.1-4.9, p=0.003), but was similar to the remitters (2.9, 2.3-3.5 *vs* 3.3, 2.7-4.3, p=NS). Additionally, TC/HDL was significantly lower in the remitters compared to the non-remitters 3.3, 2.7-4.3 *vs* 3.8, 3.1-4.9, p=0.026 **(** **Figure 4** **).**

### Factor Analysis of Lipid Parameters

Factor analysis was subsequently employed to confirm the findings in the individual lipid parameters and to stratify the groups based on their ASCVD risk. Factorization of the 7 lipid parameters **(** **Table 2** **)** yielded 2 orthogonal factors that explained 89.5% of the total variance in the original 7 lipid parameters, with communalities ranging from 0.74 to 0.99. Based on the structure of the first factor, we calculated a composite score for each subject as a weighted sum of standardized values of the original 7 lipid parameters, with much heavier weights on TC and LDL. We named this composite score as TC*LDL.

**Table 2:** Results of factor analysis of lipid parameters and factor loading with varimax rotation after adjusting for age, sex, body mass index and ethnicity.

Factor name	Factor 1	Factor 2	Communality
	TC*LDL	HDL*TG	
TC	0.98	0.12	0.98
LDL	0.97	0.06	0.94
Non-HDL	0.92	0.39	0.99
TC/HDL	0.55	0.78	0.91
HDL	0.08	-0.86	0.74
TG	0.31	0.84	0.80
TG/HDL	0.19	0.94	0.91
%Variance explained	45.3%	44.1%	89.5%

TC, total cholesterol; TG, triglycerides; HDL= high-density lipoprotein cholesterol; LDL= low-density lipoprotein cholesterol.

**Figure 5:**
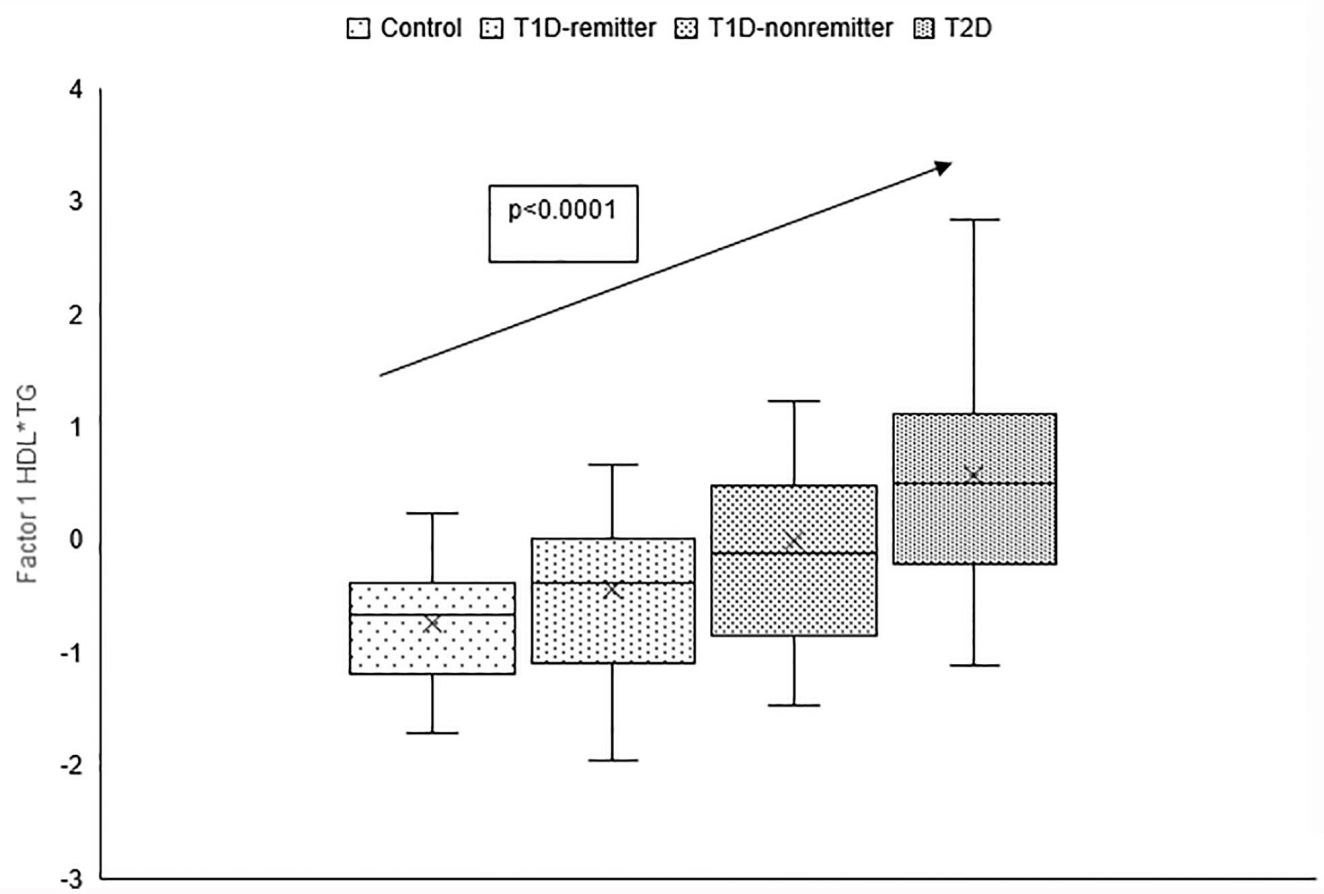
Box plots of the factorial analysis of non-HDL-C, LDL-C, TC, TC/HDL ratio designated as summary factor 1 (TC*LDL) obtained with the factor loading threshold of ≥0.45 in 203 adults. Factor 1 explained 90% of the variance in the original lipid parameters with a linear increase in mean composite scores from controls, remitters, non-remitters, and subjects with type 2 diabetes (p=0.0042).

**Figure 6:**
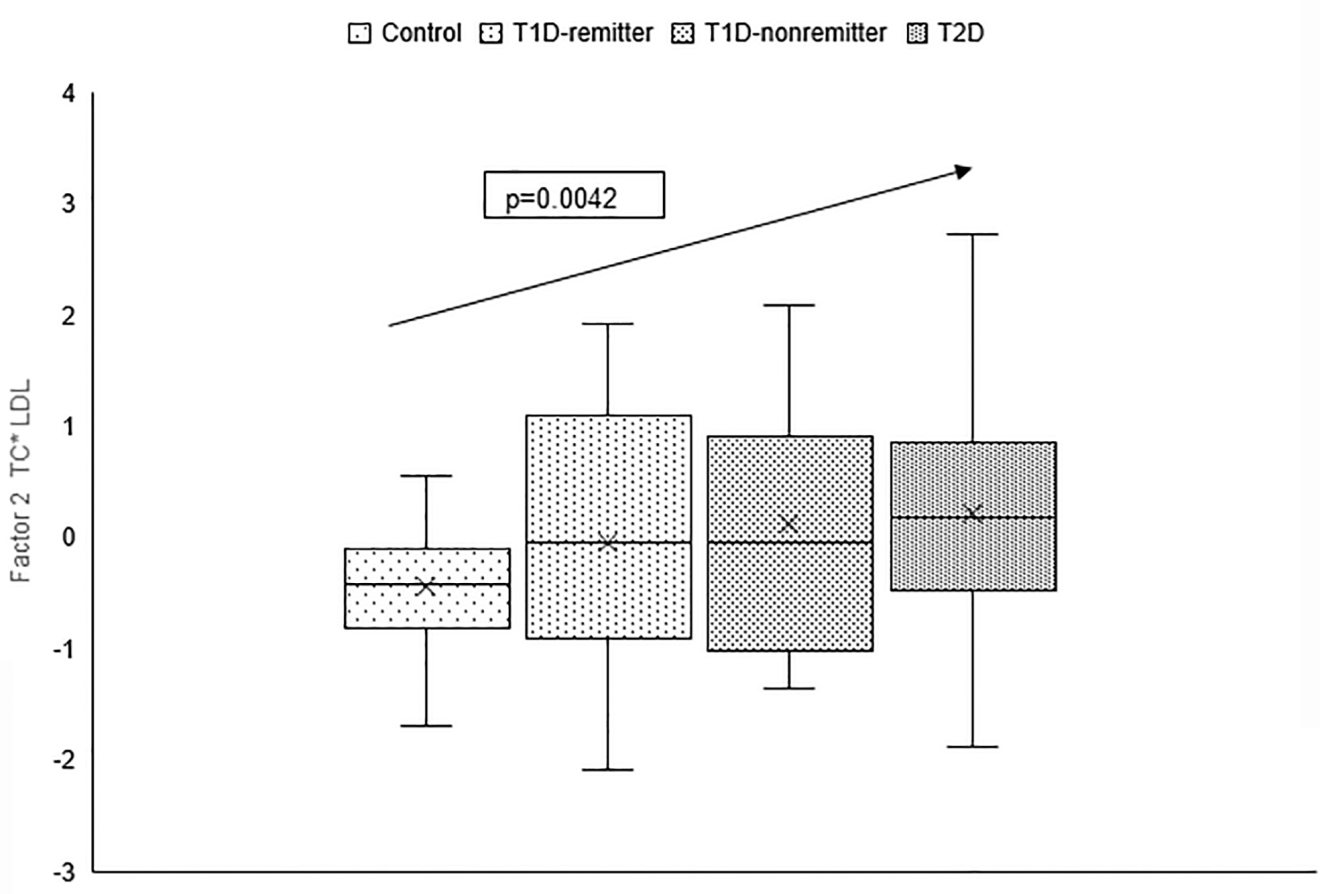
Box plots of the factorial analysis of the American Diabetes Association-recommended initial lipid parameters for the assessment of CVD risk in adults with diabetes namely, TG and HDL, and the atherogenic index of plasma, TG/HDL as designated as summary factor 2 (HDL*TG) obtained with the factor loading threshold of ≥0.45 in 203 adults. Factor 2 explained 90% of the variance in the original lipid parameters with a linear increase in mean composite scores from controls, remitters, non-remitters, and subjects with type 2 diabetes (p=0.0001).

**Figure 7:**
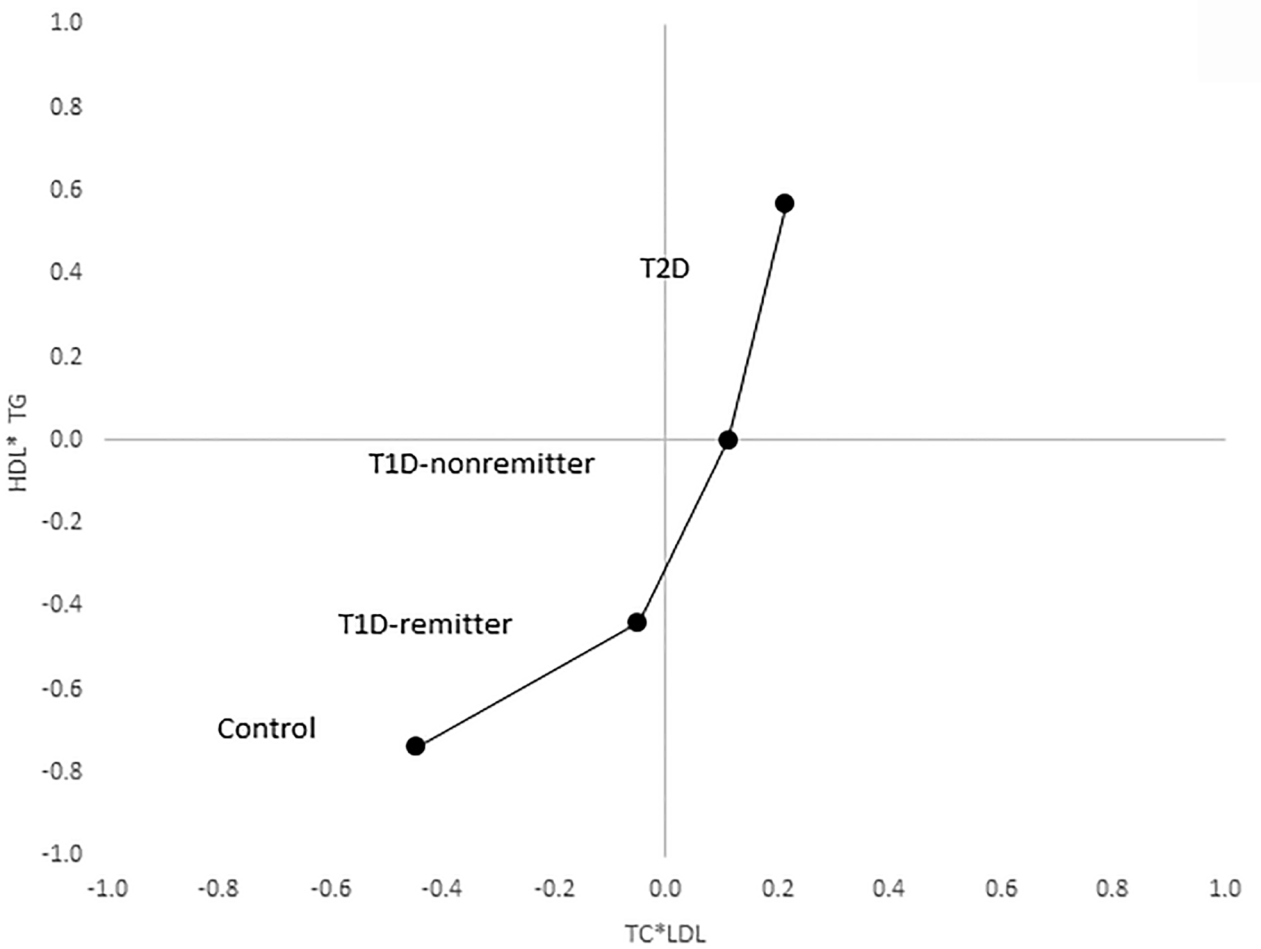
Mean composite score for lipid parameters by factor analysis. Two-dimensional depiction of the mean composite scores of factor 1 (TC*LDL) and factor 2 (HDL*TG) from the factorial analysis of the 7 lipid parameters. Both the controls and remitters are in the low composite risk quadrant, whereas non-remitters and remitters are in the higher risk quadrants. Both factor 1 and factor 2 explained 90% of the variance in the original lipid parameters, p values 0.0042 and <0.0001 respectively. The p-values for linear trends were obtained from linear regression models on composite scores, adjusted for age, sex, ethnicity and BMI.

There was a linear increase in the means of both Factor 1 (TC*LDL) and factor 2 (HDL*TG) composite scores from the control group through the remitters, non-remitters, and subjects with T2D, p value 0.0042, and <0.0001 respectively as shown in **Figures 5**, **6**. This is further illustrated in **Figure 7**, a composite two-dimensional plot showing both the controls and remitters in the low-risk quadrant, and the non-remitters and subjects with T2D in the higher-risk quadrants.

### Exploration of the Relationships Between Unstimulated C-Peptide and Lipid Parameters

There were no relationships between unstimulated C-peptide values and lipid parameters in either the remitters or non-remitters (**Supplementary Table**).

## Discussion

Risk factors for ASCVD are well established in adult patients with T2D (1), but not in their peers with T1D (6, 26). Current knowledge indicates that HbA1c, diabetic nephropathy, hypertension, and dyslipidemia are important risk factors for ASCVD in adults with established T1D (27). However, the phenotype of the earliest ASCVD risk profile at the time of diagnosis of T1D compared to T2D, and the cardinal role of PR on early lipid phenotype in T1D, which presages later ASCVD risk status, are not fully characterized. To bridge this gap, we stratified patients with newly diagnosed T1D into remitters and non-remitters and compared their earliest lipid profiles to their peers with T2D as well as a control groups to determine if there were differences in ASCVD risk profile between these T1D subtypes and T2D.

This study reports a dichotomy in the earliest, post-diagnostic, lipid-based CVD risk profile in adult patients with T1D, where the non-remitters demonstrated a similar lipid phenotype as subjects with T2D, while the remitters demonstrated a more favorable lipid profile similar to the controls. Using factorial statistics, adjusted for age, sex, race/ethnicity, and body mass index, we showed that both the controls and remitters occupy the low-risk quadrant in a two-dimensional composite risk model, while the non-remitters and subjects with T2D occupy the higher risk quadrants.

This finding of an evidence for an increased lipid-based CVD risk in adult non-remitters is consistent with our earlier study in children which showed that PR is associated with lower likelihood of early dyslipidemia in those with T1D regardless of glycemic control or the degree of adiposity at 4-5 years following the diagnosis of T1D (10). This confirmation of our earlier studies in children (10, 28, 29) points to the central role of PR on early lipid phenotypes in both children and adults with T1D that could presage a dichotomy in diabetes dyslipidemia and subsequent ASCVD risk. It also supports the hypothesis that PR could determine long-term CVD risk in T1D.

Though the mechanism of this dichotomy in lipid phenotype in T1D is unclear, we had previously proposed that the central mechanism appears to be the preservation of β-cell function and the modulatory role of residual endogenous C-peptide production in remitters on insulin resistance. Our theory that the mechanism for early dyslipidemia in non-remitters is the absence of endogenous insulin and C-peptide-mediated protection on lipid changes during puberty in adolescents (28) and adults (in this case) is consistent with the report of a protective role for C-peptide on vasculature in remitters by the Diabetes Control and Complications Trial (12). This finding has led to attempts to use exogenous C-peptide molecule in non-remitters for the prevention of vascular complications of diabetes mellitus (11).

This study demonstrates that the dichotomy in lipid profiles among the 4 groups was independent of BMI as the non-remitters, with normal BMI status, had similar lipid profile as their peers with T2D who had significantly higher BMI values. Additionally, this dichotomy was also not due to lipid-lowering therapy as subjects on lipid-lowering agents were excluded from this study. It was also not due to an optimal glycemic control as the subjects with T2D, with a moderately good A1c values at the time of diagnosis and throughout the study, had worse lipid phenotype that was similar to the non-remitters who had markedly elevated A1c values in the first year of disease. A further exploration of glycemic control during the study showed that the total daily dose of insulin was similar among the 3 groups at the time of diagnosis (p=0.08) but became significantly higher in the non-remitters at 6 months and at 12 months (p<0.0001). These findings are consistent with a pattern of worsening insulin resistance in non-remitters compared to the remitters and subjects with T2D, suggesting that an efficient endogenous C-peptide production after the diagnosis of T1D, which is present in the remitters, is necessary to maintain both optimal glycemic control and lipid profile. It is interesting that glycemic control was better in the T2D subjects who still had some endogenous C-peptide production, albeit with some associated insulin resistance (10, 28). Taken together, it appears that the dichotomy in lipid profiles among the 4 groups is driven in subjects with T1D by the presence of residual β-cell function as denoted by PR. While insulin resistance, depicted in this study by increased hemoglobin A1c and total daily dose of insulin, worsened in the non-remitters, the remitters remained insulin sensitive and required smaller doses of insulin to maintain glycemia compared to the non-remitters. These findings are consistent with our recent work in children and adolescents with diabetes mellitus where we showed that the dichotomy in lipid phenotype was not due to BMI, degree of glycemic control, or the duration of disease (10). This study is also consistent with reports showing that elevated TG/HDL ratio, the atherogenic index of plasma, is associated with increased ASCVD risk (27, 30, 31). Finally, non-HDL, the lipid parameter with a very strong correlation of 99% for increased ASCVD risk for patients with T2D (3), was significantly lower in the controls compared to the subjects with T2D and the non-remitters, but was similar between the controls and remitters.

Though these conclusions are supported by the study results, there is also the possibility of an alternative conclusion which proposes that the observed differences in the lipid profile were due to differences in the degree of insulin resistance (IR) in each group such that partial clinical remission served only as a surrogate marker of IR. This conclusion is pertinent as IR occurs in both T1D (17, 32) and T2D; and a recent study by Mock et al. reported that 55% of subjects with new-onset T1D and detectable stimulated C-peptide level of >300 pmol/L had low insulin sensitivity (i.e., high IR) and thus were not in remission when assessed by insulin-dose adjusted A1c (32). Therefore, partial clinical remission may be a marker of IR, with remitters having low IR, and non-remitters having high IR.

There are several strengths and limitations to this study. The retrospective design of this study precludes any inference to causality among the variables studied. The relatively small sample size could have impacted the results of the factor analysis. However, the consistently high communality scores confirmed the validity of the analysis. We could not confirm fasting state for all the samples used for the lipid data, however, the inclusion of non-HDL-C, which is unaffected by feeding, ensured that the results were valid as non-HDL profile was similar to those of TG and HDL which could be affected by feeding. Additionally, the use of factor statistics to create summary factors from independent lipid parameters helped address the effect of non-fasting on TG and HDL. The biological and demographic differences between T1D and T2D make it difficult to match the subjects precisely for anthropometry, race, and ethnicity. The retrospective study design also limited our ability to match the subjects prospectively. The T2D cohort was heavier and older than the T1D cohort and controls. These anthropometric factors may have resulted in a less favorable lipid profile in our T2D cohort, however, the adjustment of the factor statistics by age, sex, ethnicity, and body mass index helped address these concerns. Other potential confounders in the adult population which can affect lipid parameters include smoking, alcohol use, exercise, and thyroid dysfunction. Additionally, we did not explore the effect of some medications that are used in the adult population such as anti-viral and anti-psychotropic agents that can affect lipid levels. Though we excluded patients who were receiving statins, there are other lipid-lowering pharmacotherapies such a fibrates, ezetimibe, and fish oil that could be used in adults. However, given that these agents are much less commonly used, such a consideration should not affect the overall conclusion of this study. We used standard lipid profiles for this study, and thus did not have access to ancillary lipid parameters such as apo B and apolipoprotein CIII profiles which are also associated with increased ASCVD risk. We did not have an adequate sample size to explore the relationship between unstimulated C-peptide and lipid parameters, however, recent report indicate that stimulated serum C-peptide from mixed meal tolerance tests is a more appropriate technique for the exploration of such relationships between C-peptide and serum lipids (9). The strengths of the study include the rigorous definition of PR, the inclusion of longitudinal glycemic control data to demonstrate the impact of PR on glycemic control, and the employment of factor analysis to generate precise and comparative quantification of ASCVD risk across the groups.

In conclusion, this study reports a dichotomy in the earliest, post-diagnostic, lipid-based CVD risk profile in adult patients with T1D, such that the non-remitters demonstrate a similar lipid phenotype as subjects with T2D, while the remitters demonstrate a favorable lipid profile similar to the controls. This finding in adults is consistent with earlier reports in children and adolescents and supports the hypothesis that partial clinical remission could reduce long-term CVD risk in patients with T1D. More studies are needed to determine the possible role of partial clinical remission as a surrogate marker of insulin resistance, and how the phenomenon of early lipid dichotomy operates through the lifespan in patients with T1D.

## Data Availability Statement

The original contributions presented in the study are included in the article/**Supplementary Material**. Further inquiries can be directed to the corresponding author.

## Ethics Statement

The studies involving human participants were reviewed and approved by the University of Massachusetts Institutional Review Board. Written informed consent for participation was not required for this study in accordance with the national legislation and the institutional requirements.

## Author Contributions

All the authors have accepted responsibility for the entire content of this submitted manuscript and approved submission. BN conceived the project, researched data, and wrote the manuscript. SP researched data and helped with study design. LA-H researched data. GJ researched data. KK researched data. AL helped with study design and performed statistical analysis. All authors reviewed and approved the final version of the manuscript for submission. BN is the guarantor of this manuscript and takes full responsibility for the work as a whole.

## Funding

This study was funded in part by an investigator-initiated research grant, Grant ID: 1 R21 DK113353-03, to BN from NIDDK, NIH.

## Conflict of Interest

The authors declare that the research was conducted in the absence of any commercial or financial relationships that could be construed as a potential conflict of interest.

## Publisher’s Note

All claims expressed in this article are solely those of the authors and do not necessarily represent those of their affiliated organizations, or those of the publisher, the editors and the reviewers. Any product that may be evaluated in this article, or claim that may be made by its manufacturer, is not guaranteed or endorsed by the publisher.
