# Supplementary material for: Partial Clinical Remission Reduces Lipid-Based Cardiovascular Risk in Adult Patients With Type 1 Diabetes
Source: Front Endocrinol (Lausanne). 2021 Nov 17;12:705565. doi: 10.3389/fendo.2021.705565 (PMC8660113; doi:10.3389/fendo.2021.705565)
Supplement: Supplementary file 1 [file Table_1.docx]

|  | | | | | |
| --- | --- | --- | --- | --- | --- |
| Lipid parameter | Remitters | | Non-remitters | | p-value for Fisher's Z test |
|  | Correlation with C-peptide | N pairs | Correlation with C-peptide | N pairs |  |
| TC | -0.08 | 26 | 0.01 | 20 | 0.78 |
| LDL | -0.08 | 27 | 0.13 | 19 | 0.50 |
| HDL | 0.09 | 26 | -0.01 | 20 | 0.75 |
| TC/HDL | -0.16 | 26 | 0.09 | 20 | 0.42 |
| Non-HDL | -0.12 | 26 | 0.02 | 20 | 0.67 |
| TG | -0.16 | 26 | -0.12 | 20 | 0.90 |
| TG/HDL | -0.15 | 26 | -0.07 | 20 | 0.78 |
| Factor1: TC*LDL | -0.06 | 26 | 0.11 | 19 | 0.60 |
| Factor2: HDL*TG | -0.11 | 26 | 0.00 | 19 | 0.73 |
|  |  |  |  |  |  |

Supplementary Table 1: A Comparison of the relationships between unstimulated serum C-peptide and Lipid Parameters between Remitters and Non-remitters

TC = total cholesterol; TG = triglycerides; HDL= high-density lipoprotein cholesterol; LDL= low-density lipoprotein cholesterol
